# Integrative Analysis of miRNAs and Their Targets Involved in Ray Floret Growth in *Gerbera hybrida*

**DOI:** 10.3390/ijms23137296

**Published:** 2022-06-30

**Authors:** Yanbo Chen, Bingbing Liao, Xiaohui Lin, Qishan Luo, Xuanyan Huang, Xiaojing Wang, Qinli Shan, Yaqin Wang

**Affiliations:** 1 Guangdong Provincial Key Laboratory of Biotechnology for Plant Development, School of Life Sciences, South China Normal University, Guangzhou 510631, China; chenyanbo1221@163.com (Y.C.); liao_bbing@163.com (B.L.); lin.xhui@m.scnu.edu.cn (X.L.); luoqishan1016@163.com (Q.L.); huangxuanyan00@163.com (X.H.); wangxj@scnu.edu.cn (X.W.); 2 Guangdong Laboratory for Lingnan Modern Agricultural, Guangzhou 510642, China; 3 Flower Research Institute, Yunnan Academy of Agricultural Sciences, National Engineering Research Center for Ornamental Horticulture, Kunming 650205, China

**Keywords:** miRNA, petal growth and development, anthocyanin, *Gerbera hybrida*

## Abstract

MicroRNAs (miRNAs) are involved in regulating many aspects of plant growth and development at the post-transcriptional level. Gerbera (*Gerbera hybrida*) is an important ornamental crop. However, the role of miRNAs in the growth and development of gerbera is still unclear. In this study, we used high-throughput sequencing to analyze the expression profiles of miRNAs in ray floret during inflorescence opening. A total of 164 miRNAs were obtained, comprising 24 conserved miRNAs and 140 novel miRNAs. Ten conserved and 15 novel miRNAs were differentially expressed during ray floret growth, and 607 differentially expressed target genes of these differentially expressed miRNAs were identified using psRNATarget. We performed a comprehensive analysis of the expression profiles of the miRNAs and their targets. The changes in expression of five miRNAs (ghy-miR156, ghy-miR164, ghy-miRn24, ghy-miRn75 and ghy-miRn133) were inversely correlated with the changes in expression of their eight target genes. The miRNA cleavage sites in candidate target gene mRNAs were determined using 5′-RLM-RACE. Several miRNA-mRNA pairs were predicted to regulate ray floret growth and anthocyanin biosynthesis. In conclusion, the results of small RNA sequencing provide valuable information to reveal the mechanisms of miRNA-mediated ray floret growth and anthocyanin accumulation in gerbera.

## 1. Introduction

Petals are an important part of flowers, and are also a reference indicator for the selection of ornamental flowers [1,2,3]. Petal morphogenesis, which governs petal shape, size and color, can be divided into three stages: petal initiation, cell proliferation and cell expansion [4,5,6]. There are five main types of regulator involved in the regulatory network of petal growth and development: (1) phytohormones, including auxin, ethylene and jasmonic acid (JA); (2) transcription factors (TFs), such as CUP-SHAPED COTYLEDON1 (CUC1), CUC2, NAM/ATAF1/2/CUC2 (NAC), TEOSINTE BRANCHED1/CYCLOIDEA/PCF4 (TCP4), TCP5, AINTEGUMENTA (ANT), JAGGED (JAG) and RABBIT EARS (RBE); (3) ubiquitin pathway-related genes, which include BIG BROTHER (BB) and ubiquitin receptor DA1; (4) epigenetic regulators, such as methyltransferases or proteins involved in histone modifications; and (5) microRNAs (miRNAs) [5,7].

miRNAs are single-stranded RNAs with a length of about 19–24 nt that regulate the transcription or translation of intracellular genes at the post-transcriptional level [8]. In plants, an endogenous gene that encodes a miRNA is transcribed by RNA polymerase II to form a primary transcript (pri-miRNA), which is then cleaved twice by Dicer-like 1 (DCL1) to finally produce a mature miRNA [9,10,11]. miRNAs act by binding to RNA-induced silencing complex (RISC), but the manner of silencing is determined by the properties of the mRNAs they target [12,13]. In general, if a miRNA nearly perfectly complements an mRNA sequence, then the mRNA will be specifically cleaved by RISC; if the mRNA does not perfectly complement the miRNA, then RISC will not specifically cleave the mRNA, but only prevent translation of the mRNA into protein [13,14].

Most miRNAs are highly conserved during evolution, especially the 21 nt miRNAs, which participate in the regulation of many aspects of plant growth and development, including the morphogenesis of floral organs and anthocyanin accumulation [12,15,16,17,18,19]. The genes encoding NAC domain-containing TFs, *CUC1* and *CUC2*, are involved in boundary formation and regulating cell proliferation and are targets of miR164, which controls petal number by regulating the expression of *CUC1* and *CUC2* [20,21,22]. Almost all members of the CIN class of the TCP TF family contain miR319 target sites [23]; for example, miR319 is involved in regulating stamen and petal development by regulating the expression of *TCP4* [24]. TCP4 reduces cell proliferation by negatively regulating *GROWTH-REGULATING FACTOR* (*GRF*); it achieves this by increasing levels of miR396, which then targets GRF transcripts [25]. In *Arabidopsis* flowers, *TCP4* and *GRF* are regulated by the TF JAG, suggesting that TCP4 and GRF have similar functions in petals [25,26,27]. The conserved miRNA-mRNA pair, miR156-*SPL*, participates in various processes of plant growth and development, including juvenile-to-adult phase transition [28], flowering control [29,30], as well as anthocyanin biosynthesis [16,19]. In addition, miR858, which usually targets R2R3-MYB TF (*MYB11*, *MYB12* and *MYB111*) mRNAs, affects anthocyanin accumulation by regulating the expression of chalcone synthase (*CHS*), chalcone isomerase (*CHI*) and flavanone 3-hydroxylase (*F3H*) [31,32].

In addition to conserved miRNAs, there are many more non-conserved miRNAs in plants [17]. Some of these non-conserved miRNAs are specifically expressed in certain plant species or expressed at comparatively low levels, which makes it difficult to identify them by traditional experimental approaches [33,34,35]. An increasingly exploited approach, however, is the rapidly developing next-generation sequencing technology, which has led to the establishment of various databases holding relevant information about miRNAs; in turn, these databases provide an opportunity to identify novel miRNAs in plants. For example, miRbase (v22.1) is a relatively comprehensive database with annotation information that contains more than 48,000 mature miRNAs from 271 organisms [36].

The Asteraceae (Compositae) family is one of the largest in the world, with about 1600 genera and 22,000 species, including many edible, medicinal and ornamental plants (e.g., lettuce, chicory, safflower, daisy, dandelion, sunflower, chrysanthemum, gerbera) [37,38,39]. Nevertheless, the characterization of Asteraceae family miRNAs remains limited. To date, miRNA information from several members of this family, such as *Cynara cardunculus* (57), *Helianthus tuberosus* (16), *H. annuus* (7), *H. petiolaris* (3), *H. paradoxus* (3), *H. ciliaris* (3) and *H. exilis* (2), is collected in miRbase.

Gerbera (*Gerbera hybrida*), a member of the Asteraceae family, is world-renowned as an ornamental flower; it possesses significant commercial value in the flower market because of its colorful and highly diverse inflorescence [40,41]. There are a number of recent studies on the molecular regulation of inflorescence development and ray petal (petals of ray florets) elongation in gerbera, with a focus on organ determination, cell proliferation and cell expansion. Environmental signals (light), phytohormones (abscisic acid, brassinosteroid, gibberellin, auxin and ethylene), TFs including LEAFY (LFY), Gerbera Regulator of Capitulum Development 1–8 (GRCD1–8), GhCYC2/3/4/5, Gh-SOC1, Gerbera SQUAMOSA-LIKEs (GSQUAs), GhWIP2, GhMIF, GhEIL1) and other regulatory genes (*Gerbera hybrida* homolog of the gibberellin [GA]-stimulated transcript 1 [GAST1] from tomato (*GEG*) and (Proline-rich GASA-like) from gerbera (*PRGL*)) are all involved in regulating the growth and development of gerbera inflorescences [42,43,44,45,46,47,48,49,50,51,52,53,54,55,56,57,58]. However, which miRNAs are involved in this process has not yet been investigated.

In this study, we examined petal morphology and the petal cells of ray florets at different growth stages in gerbera. Furthermore, we analyzed the differentially expressed miRNAs during ray floret growth and found some miRNAs and their target genes related to the ray floret growth of gerbera. It provides insight into the mechanism of miRNA-mediated ray floret growth and anthocyanin accumulation in gerbera. This study also highlights a number of candidate miRNAs and their target genes for gerbera breeding.

## 2. Results

### 2.1. Phenotypic Characterization of Ray Florets during Inflorescence Opening

**Figure 1:**
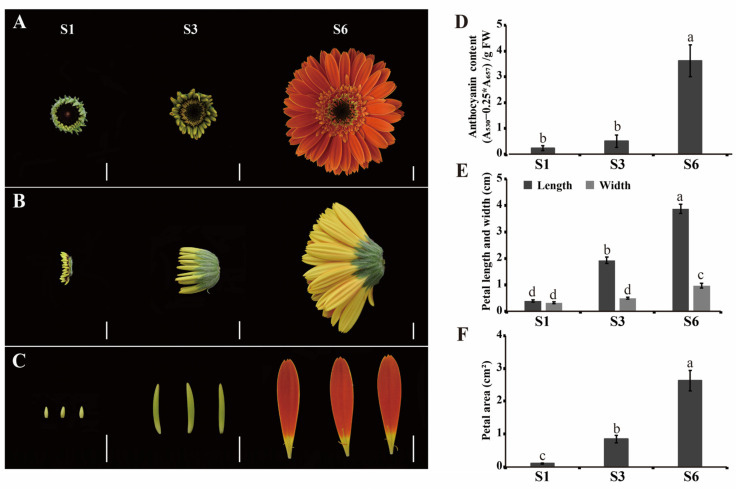
Morphological features of ray florets at different growth and development stages. (**A**–**C**), Front (**A**) and side (**B**) view of inflorescence, and ray florets (**C**) of gerbera at different stages. Scale bar = 1 cm. (**D**–**F**), The anthocyanin contents (**D**), length and width (**E**), and area (**F**) of ray petals at different stages. FW: fresh weight. Statistical significance is indicated by different letters (*p* < 0.05). Values represent means of *n* = 3 ± SD.

Gerbera is a perennial plant in the Asteraceae family with important ornamental value, and the outermost whorl of ray florets is the most conspicuous part of the gerbera capitulum. The most obvious changes during the opening of the inflorescence are the size and color of the petals (Figure 1A–C). We measured the anthocyanin content of ray florets at different stages and showed that the anthocyanin content was low at S1 and S3, but present in significant amounts at S6 (Figure 1D). In addition, we measured the length, width and area of ray petals at different growth stages, and found that the length of the petals increased significantly, while the petal width changed slightly with increasing petal area during inflorescence growth (Figure 1E). These results suggest that the growth of ray florets can mainly be attributed to the elongation of petals (Figure 1A–C,E,F).

**Figure 2:**
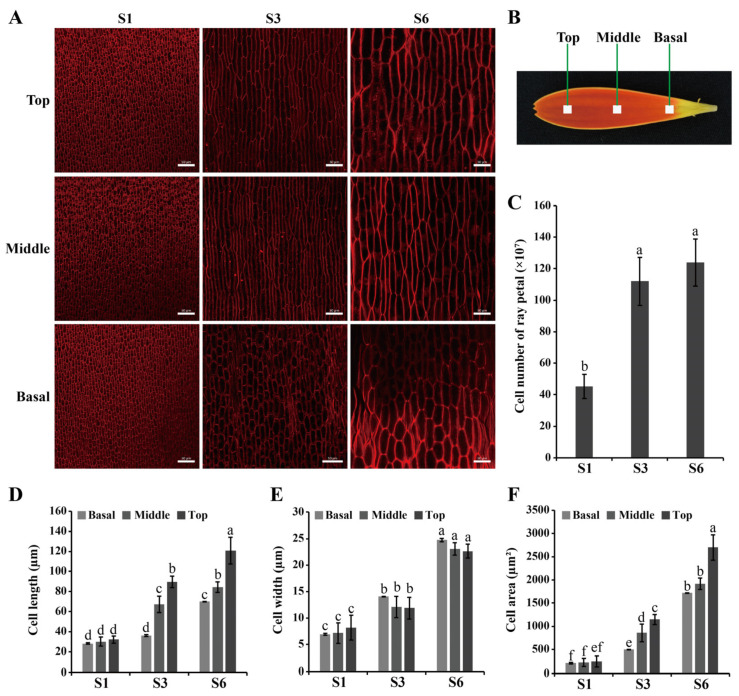
Comparison of cell size, cell number and cell area in each region of developing ray petals. (**A**) Morphological characterization of petal cells. Scale bar = 50 µm. (**B**) Blocks (1 mm^2^) at the center of the basal, middle or top regions of the ray florets were sampled for morphological characterization of petal cells. (**C**) Cell number of ray petals. (**D**–**F**), The cell length (**D**), cell width (**E**) and cell area (**F**) in each region of ray petals at different stages. Statistical significance is indicated by different letters (*p* < 0.05). Values represent means of *n* = 3 ± SD.

The morphology and number of ray petal cells at different growth and developmental stages were assessed using laser confocal microscopy (Figure 2). We found that the number of petal cells increased significantly from S1 to S3, while the petal cell number changed only slightly from S3 to S6 (Figure 2C). We also measured the cell length, width and area in different regions of the ray petals and at different stages: all three parameters gradually increased as growth progressed, and in all parts of the petal, i.e., top, middle and basal (Figure 2A,B,D–F). The length and width of the cells did not show significant differences among the various parts of S1 petals (Figure 2D,E). However, in S3 and S6 ray petals, the length of all cells gradually increased from the basal to the top, although there was no significant difference in cell width in any part of the petal (Figure 2D,E). Taken together, these results indicate that the elongation of ray petals from S1 to S3 is mainly attributed to cell proliferation, while petal elongation from S3 to S6 is mainly driven by cell expansion (Figure 1 and Figure 2).

### 2.2. Identification of Conserved and Novel miRNAs in Gerbera

To investigate how many miRNAs are involved in the growth and development of gerbera ray petals, small RNA sequencing (sRNA-seq) was carried out using ray florets from different stages. After removing adaptor reads, tRNA, rRNA, snRNA and sequences shorter than 18 or longer than 30 nt, an average of 4.33 million unannotated reads from all nine libraries (representing three biological replicates of S1, S3 and S6 ray florets) were used for further analysis. As no reference genome is available for gerbera, unannotated reads of sRNAs were mapped onto a gerbera reference transcriptome using Bowtie software to obtain mapped reads. A total of 5,825,425 mapped reads were obtained for the next step in the analysis.

**Figure 3:**
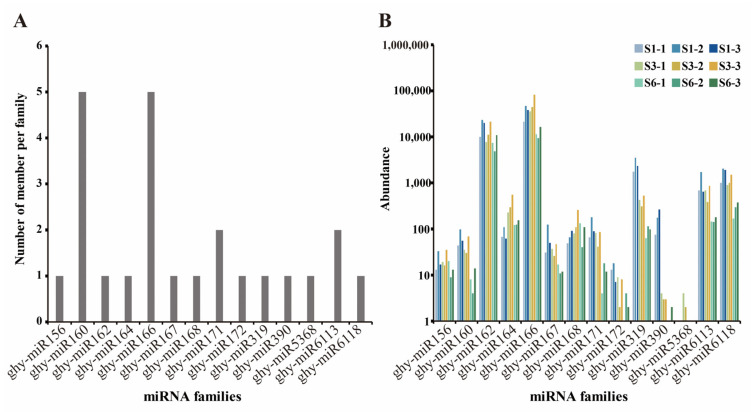
Identification of conserved miRNA families from gerbera. (**A**) Distribution of conserved miRNA family members. (**B**) Counts of each conserved miRNA family. S1, S3, and S6 represent ray petals at different stages, and the numbers after the hyphen represent different biological replicates.

For conserved miRNA identification, we compared the mapped reads with the mature sequences of conserved miRNAs in the miRBase (v22.1) database, which allows up to two mismatches with E-value < 0.01 [59]; matching reads were considered to identify conserved miRNAs. A total of 24 conserved miRNAs with precursor sequences belonging to 14 miRNA families were obtained (Supplementary Table S1). The number of representatives of various conserved miRNA families was counted (Figure 3A). In addition, the distribution and read counts for each conserved miRNA family were analyzed. The results indicate that miRNA families were present at significantly different abundance, with the ghy-miR166 family showing the highest abundance, followed by the ghy-miR162, ghy-miR6118, ghy-miR6113 and ghy-miR319 families (Figure 3B).

**Figure 4:**
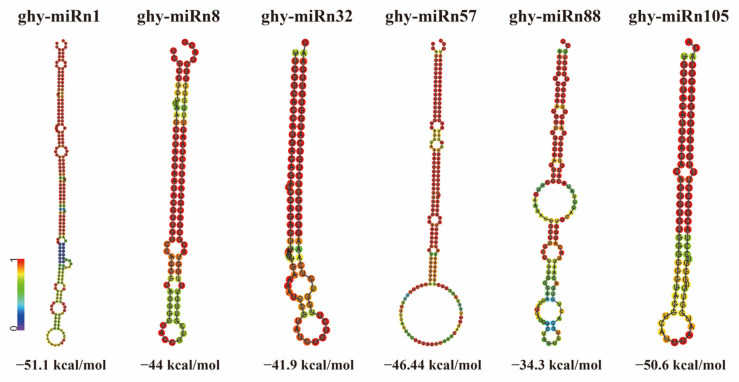
Predicted stem-loop structures of six novel miRNAs identified in gerbera. The stem-loop structures are colored by base-pairing probabilities, red: high probability, purple: low probability. kcal/mol: the minimum free energy of the stem-loop structures.

For novel miRNA identification, we used the miRDeep2 software to obtain possible precursor sequences by comparing the reads to the position information in the transcriptome. Based on the distribution information of the reads on the precursor sequences (including the position of mature sequence, star sequence and loop) and the energy information of the precursor structures, and using a Bayesian model for scoring, a total of 140 novel miRNAs and their precursors were discovered in the nine miRNA libraries (Supplementary Table S2). The length ranges of the mature sequences and hairpin sequences of the novel miRNAs were 18–24 nt and 64–250 nt, respectively. Most of the novel miRNAs have mature and hairpin sequence lengths of 24 nt and 250 nt, respectively. The minimum free energy (MFE), which indicates the stability of the hairpin structures, was between −139.8 and −15.5 kcal/mol according to RNAfold. The predicted precursor sequences and hairpin structures of the novel miRNAs are presented in Supplementary Table S2. The predicted stem-loop structures of six randomly selected novel miRNAs candidates are presented in Figure 4 as examples.

### 2.3. Differential Expression of miRNAs during the Growth of Ray Floret

**Figure 5:**
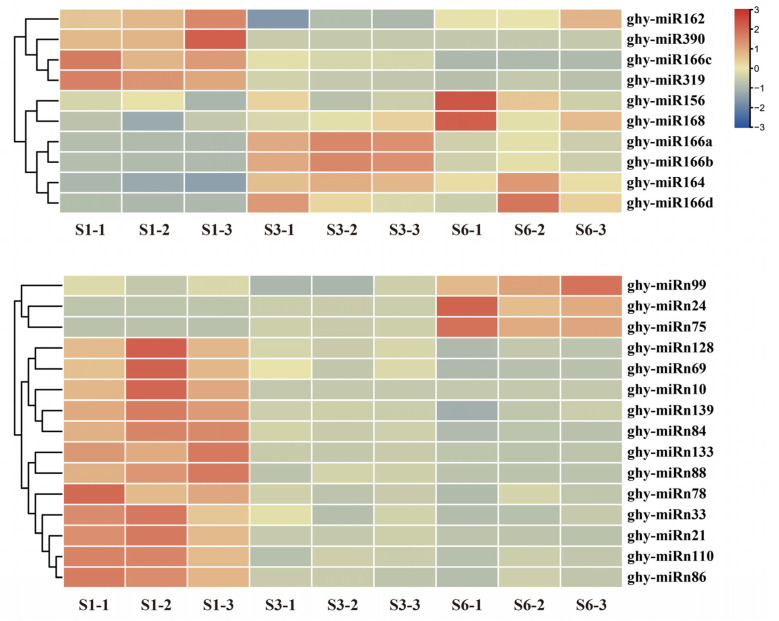
Heat map of differentially expressed miRNAs in gerbera. The miRNAs were clustered by hierarchical clustering according to their expression patterns during growth of ray florets. The expression levels are marked by color with red indicating upregulation and blue indicating downregulation.

To identify miRNAs relating to petal growth and anthocyanin accumulation in gerbera, the expression levels of the 24 conserved miRNAs and 140 novel miRNAs were analyzed. A total of 25 miRNAs, comprising 10 conserved miRNAs and 15 novel miRNAs, showed highly significant differences in expression during petal growth (using the criteria |log2(FC)| ≥ 1 and *p*-value ≤ 0.05) (Supplementary Table S3). Cluster analysis of differentially expressed miRNAs (DEMs) was performed and is shown as a heat map (Figure 5). These miRNAs are characterized by different expression patterns at S1, S3 and S6.

The majority of the miRNAs (16 of 25), comprising four conserved miRNAs and 12 novel miRNAs, exhibited high expression levels at S1, and were downregulated during ray floret growth. Only ghy-miR166c of the ghy-miR166 family displayed consistently decreased expression, while exhibiting expression peaks at S1. Like ghy-miR166c, ghy-miR319 and ghy-miR390 showed the highest expression levels at S1. Additionally, the expression levels of 12 novel miRNAs, comprising ghy-miRn10, ghy-miRn21, ghy-miRn33, ghy-miRn69, ghy-miRn78, ghy-miRn84, ghy-miRn86, ghy-miRn88, ghy-miRn110, ghy-miRn128, ghy-miRn133 and ghy-miRn139, gradually decreased with the growth of ray florets. By contrast, four conserved miRNAs (ghy-miR156, ghy-miR164, ghy-miR166d and ghy-miR168) and three novel miRNAs (ghy-miRn24, ghy-miRn75 and ghy-miRn99) exhibited elevated expression as growth progressed from S1 to S6. In addition, two DEMs with fluctuating transcription levels were found, such as ghy-miR166a and ghy-miR166b.

**Figure 6:**
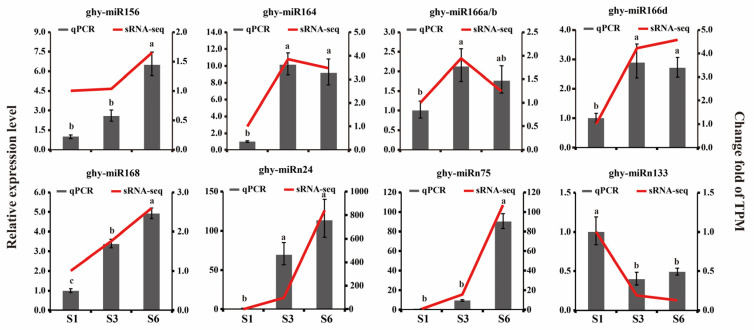
Validation of the expression profiles of DEMs at three growth stages of ray florets identified by sRNA-seq using qRT-PCR. Dark gray columns represent the relative expression levels of DEMs testing by qRT-PCR; the red lines indicate the change fold of transcripts per million mapped reads (TPM). Relative expression was normalized to the reference genes small nuclear RNA (snRNA) U6 and the expression level of S1 was defined as “1”, different letters indicate significant differences of qRT-PCR data (*p* < 0.05).

To validate the expression patterns identified by sRNA-seq, nine DEMs (six conserved miRNAs: ghy-miR156, ghy-miR164, ghy-miR166a/b, ghy-miR166d and ghy-miR168; and three novel miRNAs: ghy-miRn24, ghy-miRn75 and ghy-miRn133) were randomly selected for expression analyses in ray florets using qRT-PCR. The results showed essentially similar trends in both the qRT-PCR and sRNA-seq data (Figure 6).

### 2.4. Expression Profiles of miRNA Target Genes in Gerbera

**Figure 7:**
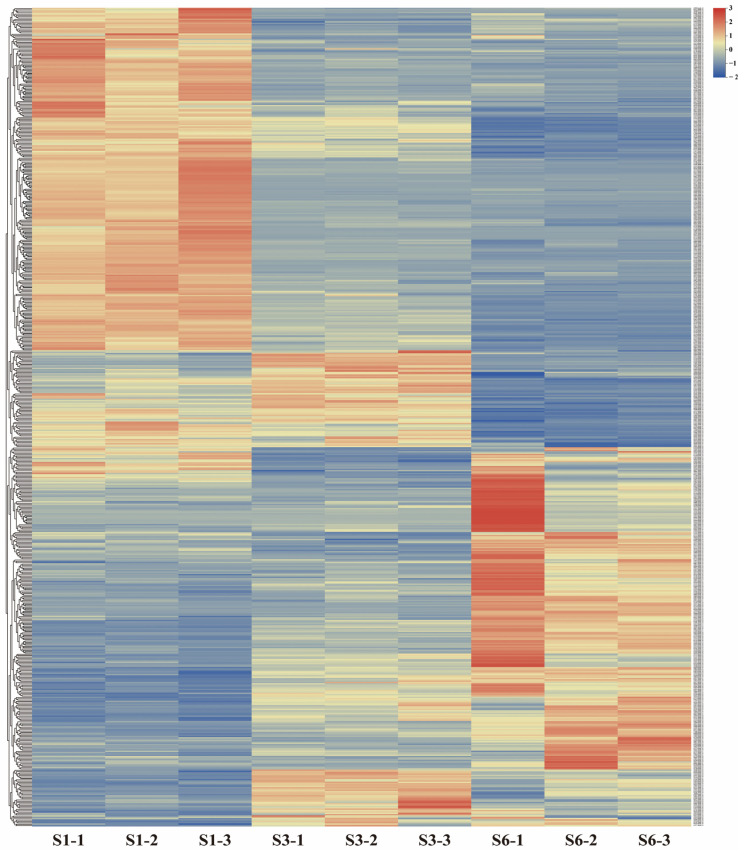
Expression patterns of 607 differentially expressed target genes of differentially expressed miRNAs in S1, S3, and S3 of ray florets. The target genes were clustered by hierarchical clustering according to their expression patterns during growth of ray florets. The expression levels are marked by color with red indicating upregulation and blue indicating downregulation.

To explore the possible biological roles in gerbera of the identified miRNAs, we computationally predicted binding sites of 164 miRNAs to transcriptome datasets using psRNATarget. With a final expectation score ≤ 5.0, 6981 candidate target genes with annotation in the GenBank nr database were identified (Supplementary Tables S4 and S5), out of which 2539 were differentially expressed hits. Only one of the 164 miRNAs, ghy-miRn66, did not predict any target gene. To identify miRNA-mRNA pairs that are crucial for gerbera ray floret growth, we focused on 607 DEGs that were identified as candidate target genes for 25 DEMs (Supplementary Table S6). The expression patterns of these candidate targets at different growth stages are visualized as a heat map (Figure 7).

In this study, we observed that the growth of ray petals is mainly due to elongation caused by cell proliferation during S1 to S3, and cell expansion during S3 to S6 (Figure 1 and Figure 2). Consistent with this, our data reveal that, during the growth of ray florets, some candidate target genes mainly related to floral organ initiation/cell proliferation and cell expansion were differentially expressed. For instance, floral organ initiation/cell proliferation-related genes downregulated at S6 were associated with organ boundary identity, meristem maintenance, growth-regulating factor, cell proliferation and axial regulator YABBY (Supplementary Table S7). In addition, cell expansion-related genes upregulated at S6 were associated with cellulose synthase, callose synthase and pectin methyltransferase (Supplementary Table S7).

Our previous studies found that phytohormones can regulate the growth of ray florets. For example, gibberellins and brassinosteroids (BRs) positively regulate ray petal elongation, while ethylene and abscisic acid (ABA) inhibit ray floret growth [49,51,55,56]. Interestingly, several phytohormone-related genes also displayed a divergent expression pattern during ray floret growth. These included BR-related genes, JA-related genes, ABA-related genes and ethylene-responsive TF (Supplementary Table S7).

Furthermore, a few differentially expressed candidate target genes associated with petal color were also identified. These include SQUAMOSA Promoter-binding Like (SPL) TF genes, a 4-coumarate-CoA ligase gene and a carotenoid biosynthesis-related gene (Supplementary Table S7). In conclusion, our results suggest that these differentially expressed candidate target genes may be involved in the growth of ray florets and the formation of petal color.

### 2.5. Validation of miRNA and Target Gene Expression

According to previous studies, NAC TFs (e.g., CUC) and SPL TFs, respectively regulated by miR164 and miR156, function as positive regulators of petal initiation/cell proliferation and negative regulators of anthocyanin synthesis, respectively [5,16,60]. In addition, callose synthase and ARF TFs are also involved in cell proliferation and cell expansion, respectively [61,62,63,64]. In this study, five NAC TFs were paired with ghy-miR164, five SPL TFs were paired with ghy-miR156, three ARF TFs were paired with ghy-miR160, and callose synthase was paired with ghy-miRn13 (Supplementary Tables S4 and S5), suggesting that these miRNAs may be involved in ray petal growth and anthocyanin accumulation by regulating their respective targets.

**Figure 8:**
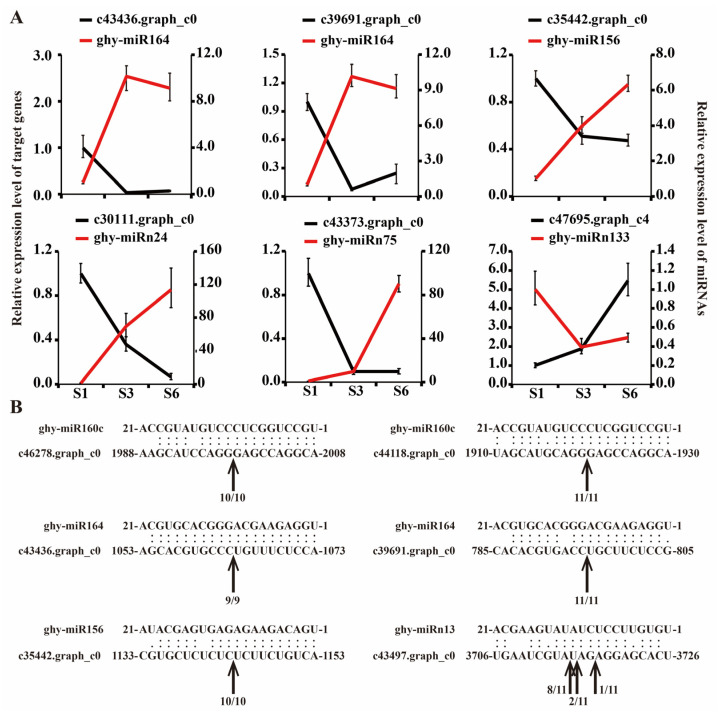
Validation of miRNA predicted targets. (**A**) Expression of eight candidate miRNA-mRNA pairs using qRT-PCR. Relative expression levels of miRNAs and candidate target genes were normalized to the reference genes snRNA U6 and *GhACTIN* (GenBank accession number AJ763915), respectively. The expression level of S1 was defined as “1”. (**B**) Cleavage sites identified by 5′-RLM-RACE assay in ray petals. Positions of the cleavage sites are indicated by arrows with the proportion of sequenced clones.

To verify the expression profiles of miRNAs and their target genes and to characterize the predicted cleavage sites, we performed qRT-PCR and 5′-RLM-RACE. Eight miRNA-mRNA pairs displayed negative correlations in their expression patterns (Figure 8A). The cleavage sites for three pairs were verified by 5′-RLM-RACE and their positions were generally consistent with the predicted results (Figure 8B): the cleavage sites for these miRNA-mRNA pairs were between positions 10 and 11 of the miRNA binding site, which is the canonical position for cleavage by AGO [13,65]. Nevertheless, we also identified some non-canonical cleavage positions. For instance, for ghy-miRn13 pairing with the c43497.graph_c0 transcript, the cleavage site was located at positions 9–10, 11–12 and 12–13, which is similar to those reported for the miR408-PCY miRNA-mRNA pair in Arabidopsis [66]. Although ghy-miR160 did not show differential expression during ray petal growth, we verified the cleavage sites in its ARF target genes by 5′-RLM-RACE and found these to be consistent with predictions. Thus, our findings suggest that the miRNAs mentioned above may regulate ray petal growth and anthocyanin accumulation by cleavage of target gene mRNAs.

## 3. Discussion

### 3.1. Conserved and Novel miRNAs in Gerbera Ray Petals

In this study, small RNA sequencing was performed on the growing ray petals of gerbera. Bioinformatics identified 24 conserved and 140 novel miRNAs together with precursor sequences using a gerbera transcriptome. The 24 conserved miRNAs can be categorized into 14 miRNA families. The transcripts per million mapped reads (TPM) of these conserved miRNAs varied from 19.3 (ghy-miR5368) to 330,150 (ghy-miR166e), suggesting that the expression patterns of the various miRNAs are extremely different. Five miRNA families (ghy-miR162, ghy-miR166, ghy-miR319, ghy-miR6113 and ghy-miR6118; comprising 10 individual miRNAs) are highly expressed (TPM ≥ 10,000) at S1, S3 or S6, which is similar to previous results in *Carya cathayensis* [67] and *Osmanthus fragrans* [68]. In addition, eight conserved miRNA families, i.e., ghy-miR156, ghy-miR160, ghy-miR164, ghy-miR167, ghy-miR168, ghy-miR171, ghy-miR172 and ghy-miR390, showed moderate expression levels (TPM between 100 and 10,000) at S1, S3 or S6. Even though there are 14 conserved miRNAs (highly/moderately expressed at S1, S3 or S6) that do not show differential expression during petal growth in gerbera, they do seem to be essential for plant growth and development according to previous studies in other plants. For instance, miR160 positively regulates the length of cotton fiber [69], hypocotyl elongation in *Arabidopsis* [64], and blade outgrowth and floral organ development in tomato [63] by downregulating its target *ARF* genes. The targets of miR167 also include *ARF* genes, which are involved in female sterility and flower development [70,71,72], the growth and development of rice [73], and inflorescence stem elongation [74]. miR171 regulates chlorophyll biosynthesis [75] and floral meristem identity [76]. miR172 is another highly conserved miRNA that plays an important role in floral organ identity, sex determination and flowering time [77,78,79,80]. Even miRNAs that are expressed at very low levels in gerbera ray petals, such as ghy-miR5368, which has a TPM ranging from 0 to 66.6, may have an impact on growth and other biological processes. For instance, miR5368 is involved in regulating growth of *Picrorhiza kurroa* [81] and in the drought response of alfalfa [82].

In this work we identified 140 predicted novel miRNAs, whose hairpin structures mapped to the gerbera transcriptome. There were six novel miRNAs with a TPM > 10,000 at one of the three floral growth and development stages studied, i.e., ghy-miRn8, ghy-miRn24, ghy-miRn32, ghy-miRn75, ghy-miRn102 and ghy-miRn131. In addition to these, 68.6% (96/140) of the novel miRNAs showed moderate expression levels (TPM between 100 and 10,000) at S1, S3 or S6. Nevertheless, this still left 38 novel miRNAs that were expressed at low abundance. Therefore, the 102 predicted novel miRNAs that show high or moderate expression levels are the focus of our future research.

### 3.2. Correlation Analysis of Differentially Expressed miRNAs and miRNA Target Genes

To discover and unravel the roles of miRNAs in regulating the growth of gerbera petals, it is crucial to analyze miRNAs together with their potential target genes. We identified 13,138 candidate target genes using psRNATarget with a final expectation score ≤ 5, out of which 6981 genes matched protein sequences in the GenBank nr database. However, 30.5% (534/1751) and 48.5% (5737/11,836) of the predicted targets of conserved and novel miRNAs, respectively, did not have orthologs in the GenBank nr database, implying they might be novel genes in gerbera.

Differentially expressed target genes of DEMs were a priority of our investigation. Using psRNATarget, 607 DEGs were paired with 25 DEMs in this study. GO analysis of the 607 DEGs suggested their possible involvement in multiple biological processes, molecular functions and cellular components (Supplementary Figure S1). The biological processes mostly related to macromolecule metabolic process (51), cell growth (41), regulation of transcription (38), small molecule biosynthetic process (29) and catabolic process (27). The major cellular components for these target genes were classified as integral component of membrane (188), nucleus (61), cytoplasm (50), chloroplast (15) and plastid (8). For molecular functions, the GO terms centered on ATP binding (92), protein kinase activity (50), DNA binding (41), protein binding (25) and catalytic activity (21).

Interestingly, some of the potential target genes of both conserved and novel miRNAs were important for ray floret growth and development. For instance, miR164 paired with *CUC1* and *CUC2*, and miR319 paired with *TCP4* all play an important role in petal initiation/cell proliferation [5]. *CUC1* and *CUC2*, belonging to the NAC TF family, are specifically expressed at boundaries and are essential for petal organogenesis [60,83,84]. Interestingly, miR164 was also found to be ethylene-responsive, regulating cell expansion in rose petals [17]. As shown in Figure 8A, two NAC-domain TFs (c43436.graph_c0 and c39691.graph_c0) showed a decreasing expression trend with ray petal growth, which was in contrast to the expression profile of ghy-miR164. In *Arabidopsis*, miR319 promotes cell proliferation by negatively regulating *TCP4* [85]. In this study, the expression level of ghy-miR319 was high during S1, but then gradually decreased as ray petals grew. These results imply that ghy-miR319 plays a role in the cell proliferation that occurs during the early stages of petal growth (S1 to S3) in gerbera. In maize leaf, three miRNAs, i.e., miR166, miR168 and miR390, are significantly upregulated in the meristem compared with the elongation and mature zones [86]. Our data partially reflect this result: ghy-miR166 and ghy-miR168 expression was upregulated during petal growth, but ghy-miR390 was gradually downregulated. miR390 typically targets the *trans-acting short interference RNA3* (*TAS3*) transcript and helps maintain polarity in leaf [87,88,89,90].

Similar to miR390, miR166 regulates class III homeodomain leucine-zipper proteins and plays a role in meristem formation and leaf polarity [91,92,93,94]. Both leaves and petals are lateral organs, which undergo cell proliferation and cell expansion to establish polarity and eventually reach a final shape and size [95]. Although there are no published studies on the regulation of petal growth by miR166 and miR390, we speculate that these two functionally conserved miRNAs maybe have similar roles in the petal growth of gerbera. BIM1, a basic helix-loop-helix (bHLH) TF, usually interacts with BES1/BZR1 to synergistically regulate the expression of many BR-induced genes by binding to the E box (CANNTG) sequence of these gene promoters [96]. In gerbera, BR plays a positive role in petal growth by stimulating the elongation of petal cells [55]. In this work, *BES1/BZR1* (c49939.graph_c1) was predicted to be the target genes of ghy-miRn133. The target gene showed the opposite expression trend to ghy-miRn133, although the predicted effect of ghy-miRn133 on *BES1/BZR1* (c49939.graph_c1) is translational repression. In addition, *GRCD4* (c42659.graph_c0) was predicted to be a target of ghy-miRn75, which is dramatically upregulated during petal growth. *GRCD4* is functionally redundant with *GRCD5*; both provide an E function in floral development, with a role in organ determination in gerbera [45]. Our data show that the fold change of *GRCD4* (c42659.graph_c0) mRNA during petal growth was less than two, probably because it is regulated by other factors besides miRNAs.

The anthocyanin content of ray petals gradually increases as they progress from S1 to S6 and reaches its highest level at S6. We found several miRNAs relating to anthocyanin biosynthesis that are differentially expressed during petal growth, including ghy-miR156, ghy-miR164, ghy-miRn24 and ghy-miRn75. In *Arabidopsis*, miR156 positively regulates anthocyanin accumulation by cleaving the mRNA of *SPL9*, which blocks anthocyanin biosynthesis by disrupting the formation of the MYB-bHLH-WD40 (MBW) transcriptional activation complex [16]. In this study, ghy-miR156 accumulated to high levels during S6, which implies an association with anthocyanin biosynthesis. In addition, miR164-encoded peptides (miPEP164), which are encoded by primary transcripts of miR164, positively regulate the proanthocyanidin biosynthetic pathway [97]. In this study, levels of the mature sequence of ghy-miR164 were shown to increase with ray petal growth, and thus it will be interesting to investigate the role of miPEP164 in the anthocyanin biosynthesis pathway in gerbera ray petals. MYB5, a R2R3-MYB TF, is a negative regulator of the phenylpropanoid/flavonoid synthesis pathway [98]. Here, we found *MYB5* (c46137.graph_c1) to be a candidate target gene for ghy-miRn24. Although ghy-miRn24 shows dramatic upregulation during petal growth, the expression of *MYB5* (c46137.graph_c1) does not change significantly. In addition, a gerbera gene, *GMYC1* (c39028.graph_c0), is also predicted to be a target of ghy-miRn75, a miRNA that is dramatically upregulated during petal growth. GMYC1 is a bHLH-type TF that interacts with GMYB10 and activates *PGDFR2* by binding to its promoter; in turn, *PGDFR2* regulates the biosynthesis of anthocyanins [99,100]. Similar to *GRCD4* (c42659.graph_c0), our data indicate that the fold change of *GMYC1* (c39028.graph_c0) was less than two during petal growth, which is possibly because they are regulated by miRNAs together with other factors. Taken together, our results suggest that the conserved and novel miRNAs we identifiy in this study may be involved in regulating floral organ growth and anthocyanin biosynthesis, and will be the subject of further investigations.

## 4. Materials and Methods

### 4.1. Plant Materials

*Gerbera hybrida* (cultivar ‘Shenzhen No. 5’) plants were grown under natural light conditions in Foshan, Guangdong Province of China. Inflorescences were harvested at stage 1 (S1), S3 and S6 [101]. Cut gerbera were immediately placed in a water bottle and transported to the laboratory within 1.5 h. Each ray floret sample used for sequencing was collected from at least twenty inflorescences and frozen in liquid nitrogen immediately, then stored at −80 °C.

### 4.2. Length, Width and Size of Petals and Cells

To record changes in ray petals during the growth process, images were taken using a camera (D7200, Nikon, Japan) at different stages. For each sample, ray florets from twenty inflorescences were collected. The length, width and area of ray petals were measured using ImageJ software.

To measure length, width and area of ray petal cells, petals were fixed for 30 min in FAA (1:1:18 ratios of formalin, acetic acid and 70% ethanol by volume), and a 1 mm^2^ block at the center of three regions (top, middle and basal) was dissected and stained with 0.1 mg mL^−1^ propidium iodide for 1 h at room temperature. S1 petals were too small for dissection, so we used the whole petal instead. The adaxial epidermal cells of ray petals were imaged using a laser confocal scanning microscope (LSM710/ConfoCor2, Carl-Zeiss, Germany). Then ImageJ software was used to determine cell dimensions. More than 200 cells from 10 different inflorescence petals were randomly selected as one biological replicate and a total of three replicates to be measured.

### 4.3. Measurement of Anthocyanin Content

For the anthocyanin content, ray florets at different stages were weighed and placed in centrifuge tubes. A solution containing hydrochloric acid and methanol (*v*/*v* = 1:99) was added into the tubes and placed at 4 °C for 48 h. After centrifuging, the absorbance (A) of the extract was measured at 530 nm (A_530_) and 657 nm (A_657_) using a spectrophotometer. The total anthocyanin content was calculated using the formula (A_530_ − 0.25*A_657_)/g fresh weight [102,103].

### 4.4. Library Construction and Sequencing

For transcriptome sequencing, total RNA was extracted from nine samples of ray florets (three biological replicates of S1, S3 and S6 florets) using the NEBNext Ultra II RNA Library Prep Kit for Illumina (NEB, Code No. E7775) according to the manufacturer’s instructions. The concentration, integrity and purity of total RNA were determined using NanoDrop, Agilent 2100 and 1% agarose gel electrophoresis. Then, poly(A) mRNA was isolated from total RNA using oligo(dT)-linked magnetic beads (Vazyme, Code No. N411-01). Next, the mRNA was subjected to a series of treatments and the cDNA libraries was constructed as previously described [104]. Sequencing was conducted with the NovaSeq 6000 high-throughput sequencing platform (Illumina) at Beijing Biomarker Technologies Co. Ltd. (Beijing, China). The raw reads were trimmed by removing adaptor sequences and filtering low-quality reads, then the clean reads were assembled de novo using Trinity software. We used DIAMOND software [105] (E-value < 10^−5^) to compare unigene sequences with the NR, Swiss-Prot, COG, KOG, eggNOG 4.5 and KEGG databases and the InterProScan database was used to analyze the GO Orthology results for new unigenes. After predicting the amino acid sequences of unigenes, HMMER software (E-value < 10^−10^) was used to query the Pfam database to obtain annotation information. The assembled transcriptome data was used as the reference for sequence alignment and subsequent analysis.

For small RNA sequencing, RNA was extracted from ray florets, similar to those used for the transcriptome, using NEBNext Multiplex Small RNA Library Prep Set for Illumina (Set 1) (NEB, Code No. E7300L) according to the manufacturer’s instructions. The RNA quality was tested as described for transcriptome sequencing and nine sRNA libraries were constructed and sequenced using the SE50 high-throughput sequencing platform (Illumina).

### 4.5. Bioinformatics of miRNAs

A series of standard steps were applied to obtain clean data after small RNA sequencing, briefly described as follows: filter low-quality reads, remove adaptor sequences, exclude sequences shorter than 18 and longer than 30 nt and reads with a content of unknown bases (signified N) greater than 10%. Using Bowtie software, clean reads were compared with the Silva, GtRNAdb, Rfam and Repbase databases, and rRNAs, tRNAs, snRNAs, snoRNAs, ncRNA and repeats were filtered to obtain unannotated reads. The assembled gerbera transcriptome data were used as the reference for sequence alignment and subsequent analysis. The unannotated reads were compared against the reference transcriptome data using Bowtie software to obtain mapped reads.

For conserved miRNA identification, we matched the mapped reads with the mature sequences of known miRNAs in miRBase (v22) allowing at most two mismatches, such that matched reads were considered to be conserved miRNAs.

For mapped reads that were not identified as conserved miRNAs, we used miRDeep2 software for prediction of novel miRNAs. Possible precursor sequences were obtained by comparing reads to the assembled transcriptome data. Then, by adjusting the parameters and score system of the miRDeep2 package [106,107], we used Bayesian statistics to determine possible novel miRNAs based on the distribution of corresponding reads along precursor sequences (taking into account how miRNAs are produced and the characteristics of mature sequence, star sequences and loops) and free energy predictions (RNAfold) of precursor structures.

### 4.6. Analysis of Differentially Expressed miRNAs (DEMs)

The R package DESeq2 was used to identify DEMs across different growth stages of gerbera ray floret. miRNA fold-changes were normalized against transcripts per million mapped reads (TPM). The screening criteria for DEMs were as follows: |log2(FC)| ≥ 1 and *p*-value ≤ 0.05. DEM heat maps, which included both conserved and novel miRNAs, were generated using TBtools (v1.09867).

### 4.7. Quantitative Real-Time PCR (qRT-PCR) Analysis of Differentially Expressed miRNAs

Total RNA was extracted from ray florets at three growth stages (S1, S3, S6) using Trizol reagent (Invitrogen, Code No. 15596-026) according to the manufacturer’s instructions. The Mir-X miRNA First-Strand Synthesis Kit (Clontech, Code No. 638315) was used for converting miRNAs into cDNA to enable them to be quantified by qRT-PCR. Briefly, RNAs were poly(A)-tailed using poly(A) polymerase, and then copied using a modified oligo(dT) primer and SMART MMLV Reverse Transcriptase. qRT-PCR was carried out using RealStar Green Fast Mixture (GenStar, Code No. A301-01) on a CFX96 TouchTM Real-Time PCR Detection System (Bio-Rad Laboratories, Inc., Hercules, CA, USA). Each reaction was performed with three biological repeats and three technical repeats. The small nuclear RNA (snRNA) U6 was used as the internal reference to normalize the results. DEM expression levels were calculated using the 2^−^^∆∆CT^ method [108]. The primers used for qRT-PCR are listed in Supplementary Table S8.

### 4.8. Prediction and Annotation of miRNA Target Genes

To identify candidate target genes of conserved and novel miRNAs, psRNATarget [109,110] was used with the identified miRNAs and gerbera transcriptome data. Candidate target genes with an expectation score ≤ 5.0 were considered as potential targets of miRNA. Candidates that were functionally annotated by RefSeq non-redundant protein (Nr) databases were used in the next step of the differential expression analysis. Differentially expressed genes (DEGs), normalized against fragments per kilobase million mapped reads (FPKM), were analyzed using the DESeq2 package with |log2(FC)| ≥ 1 and FDR (false discovery rate) ≤ 0.01. The DEG heatmap was generated using TBtools (v1.09867).

### 4.9. Validation of miRNA Targets

Two methods were used in this study to validate the expression levels of candidate genes targeted by miRNAs. First, qRT-PCR was used to estimate whether miRNAs show opposite expression profiles from their target genes. Total RNA was extracted from gerbera ray florets using an Easystep Super Total RNA Extraction Kit (Promega, Code No. LS1040) following the manufacturer’s instructions. Then, ReverTra Ace qPCR RT Master Mix with a gDNA Remover Kit (Toyobo, Code No. FSQ-301) was used to synthesize first-strand cDNA from ca. 3 µg total RNA. The reagents and procedures for qRT-PCR were the same as used for miRNA. Relative expression levels of candidate target genes were normalized against the reference gene *GhACTIN* (GenBank accession number: AJ763915) [104] and the expression level at S1 was defined as “1”.

The second method was 5′-RNA ligase-mediated (RLM)-RACE. Total RNA from ray florets was ligated to the 5′-adaptor sequence (5′-GCUACACUCGGUUUGCUGGCUUU GAUGAAA-3′) using T4 RNA ligase (Takara, Code No. 2050A) at 15 °C for 18 h. The ligated RNA was used to synthesize cDNA using a ReverTra Ace qPCR RT Master Mix with a gDNA Remover Kit (Toyobo, Code No. FSQ-301). Then, 0.5 µL cDNA was used as the template for PCR. The products after two rounds of PCR were gel-purified and ligated into the *pMD-18T* vector for sequencing. The primers used for 5′-RLM-RACE are listed in Supplementary Table S9.

## Data Availability

Not applicable.

## Supplementary Materials

The following are available online at: https://www.mdpi.com/article/10.3390/ijms23137296/s1.
