# Supplementary material for: Integrative Analysis of miRNAs and Their Targets Involved in Ray Floret Growth in Gerbera hybrida
Source: Int J Mol Sci. 2022 Jun 30;23(13):7296. doi: 10.3390/ijms23137296 (PMC9266715; doi:10.3390/ijms23137296)
Supplement: Supplementary file 1 [file ijms-23-07296-s001.zip › Supplementary material information.pdf]

Supplementary Figure S1. Gene ontology (GO) classification of 607 differentially expressed genes for 25 differentially expressed miRNAs. The figure shows partial GO enrichment for the genes in three categories: biological process, molecular function and cellular component.

Supplementary Table S1. Conserved miRNAs in gerbera.

Supplementary Table S2. Novel miRNAs in gerbera.

Supplementary Table S3. Significant differential expression of miRNAs in gerbera.

Supplementary Table S4. Identified and annotated target transcripts for the conserved miRNAs in gerbera.

Supplementary Table S5. Identified and annotated target transcripts for the novel miRNAs in gerbera.

Supplementary Table S6. Expression patterns of 607 differentially expressed target genes of differentially expressed miRNAs in gerbera.

Supplementary Table S7. Candidate target genes associated with ray petal growth and development in gerbera.

Supplementary Table S8. The primers used for qRT-PCR.

Supplementary Table S9. List of primers used for 5'-RLM-RACE.
