# Supplementary figures and images for: Integrative Analysis of miRNAs and Their Targets Involved in Ray Floret Growth in Gerbera hybrida
Source: Int J Mol Sci. 2022 Jun 30;23(13):7296. doi: 10.3390/ijms23137296 (PMC9266715; doi:10.3390/ijms23137296)

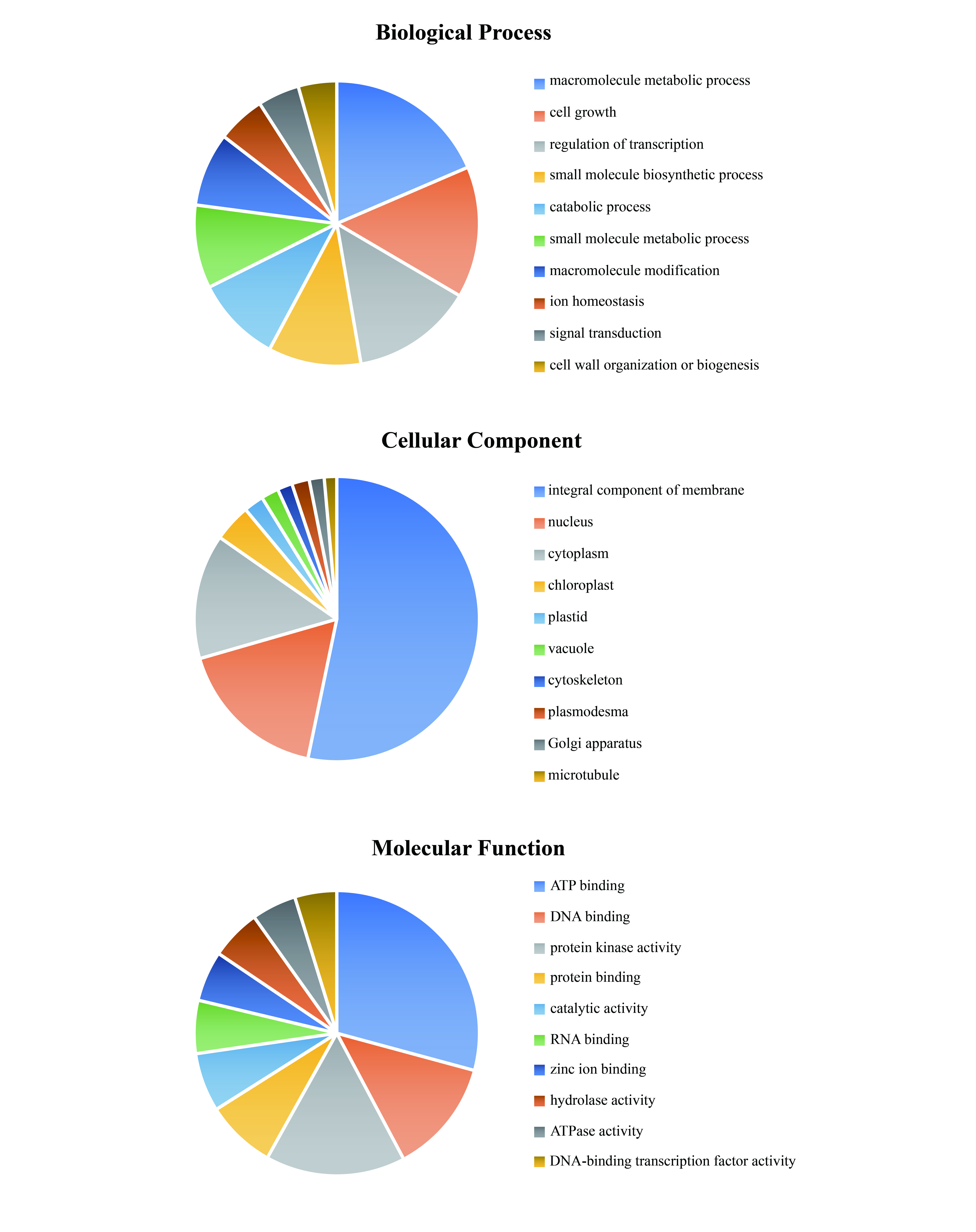

Supplement: Supplementary file 1 [file ijms-23-07296-s001.zip › Supplementary Figure 1.jpg]
